# Temporal Dissection of K-ras^G12D^ Mutant *In Vitro* and *In Vivo* Using a Regulatable K-ras^G12D^ Mouse Allele

**DOI:** 10.1371/journal.pone.0037308

**Published:** 2012-05-11

**Authors:** Zuoyun Wang, Yan Feng, Nabeel Bardessy, Kwok-Kin Wong, Xin-Yuan Liu, Hongbin Ji

**Affiliations:** 1 State Key Laboratory of Cell Biology, Institute of Biochemistry and Cell Biology, Shanghai Institutes for Biological Sciences, Chinese Academy of Sciences, Shanghai, China; 2 Massachusetts General Hospital Cancer Center, Massachusetts General Hospital, Boston, Massachusetts, United States of America; 3 Department of Medical Oncology, Dana-Farber Cancer Institute, Harvard Medical School, Boston, Massachusetts, United States of America; Cincinnati Children's Hospital Medical Center, United States of America

## Abstract

Animal models which allow the temporal regulation of gene activities are valuable for dissecting gene function in tumorigenesis. Here we have constructed a conditional inducible estrogen receptor-*K-ras^G12D^* (*ER-K-ras^G12D^*) knock-in mice allele that allows us to temporally switch on or off the activity of K-ras oncogenic mutant through tamoxifen administration. *In vitro* studies using mice embryonic fibroblast (MEF) showed that a dose of tamoxifen at 0.05 µM works optimally for activation of *ER-K-ras^G12D^* independent of the gender status. Furthermore, tamoxifen-inducible activation of *K-ras^G12D^* promotes cell proliferation, anchor-independent growth, transformation as well as invasion, potentially via activation of downstream MAPK pathway and cell cycle progression. Continuous activation of *K-ras^G12D^ in vivo* by tamoxifen treatment is sufficient to drive the neoplastic transformation of normal lung epithelial cells in mice. Tamoxifen withdrawal after the tumor formation results in apoptosis and tumor regression in mouse lungs. Taken together, these data have convincingly demonstrated that K-ras mutant is essential for neoplastic transformation and this animal model may provide an ideal platform for further detailed characterization of the role of *K-ras* oncogenic mutant during different stages of lung tumorigenesis.

## Introduction

Animal models which allows temporal regulation of gene activity have been proved very valuable for gaining insights into the gene function involved in different tumor formation stages. Previous studies have demonstrated that knock-in of ER region into certain genes including P53 and Myc to generate fusion proteins allow for precise and temporal regulation of gene activity via ectopic provision of 4-hydroxytamoxifen [1], [2]. The *Trp53ER* ^KI/KI^ mouse model is the first example of genetic models that allows specific, rapid and reversible perturbation of the biological function of a single endogenous gene *in vivo*. Studies based on this mouse model have dissected the precise role of P53 engaged in different stages of tumorigenesis as well as tumor regression [2], [3], [4], [5], [6], [7], [8]. Similarly, studies using the MycER mouse allelesignificantly contribute to our understanding about the role of Myc in cell proliferation, differentiation as well as the relationship between stem cells and cancer [1], [6], [7], [9], [10], [11], [12].

Oncogenic mutations including *K-RAS* ^G12D^ is frequently observed in approximately 20% of all types of human cancers including carcinomas of the lung, colon, and pancreas [13], [14], [15], [16], [17], [18]. K-RAS is a small GTPase which works as a binary molecular switch between a GDP-bound inactive form and a GTP-bound active form. When mutated in codon 12, 13, or 61, the K-RAS mutants remain active and constitutively transduce signals through MAPK pathway and PI3K pathway [19], [20], [21], [22]. Previous studies have demonstrated that proteins such Ras or Raf fused to ER could be activated by tamoxifen treatment [23], [24], [25], [26], [27]. However, there is no animal model has been established based on this knowledge yet.

We have here engineered a knock-in allele encoding a 4-hydroxy tamoxifen (4-OHT) inducible estrogen receptor-*K-ras^G12D^* (*ER-K-ras^G12D^*) with placing the Loxp-Stop-Loxp fragment in front of the exon 1 of *K-ras* coding region. Using the mouse embryonic fibroblast (MEF), we have shown that *ER-K-ras^G12D^* expression is induced by Adeno-Cre treatment and the activity of ER-K-ras^G12D^ mutant is regulated by an optimal dose of tamoxifen administration. We further demonstrated that the ER-K-ras^G12D^ mutant is essential for neoplastic transformation as well as tumor maintenance.

## Materials and Methods

### Mouse cohorts and Treatment

**Figure 1:**
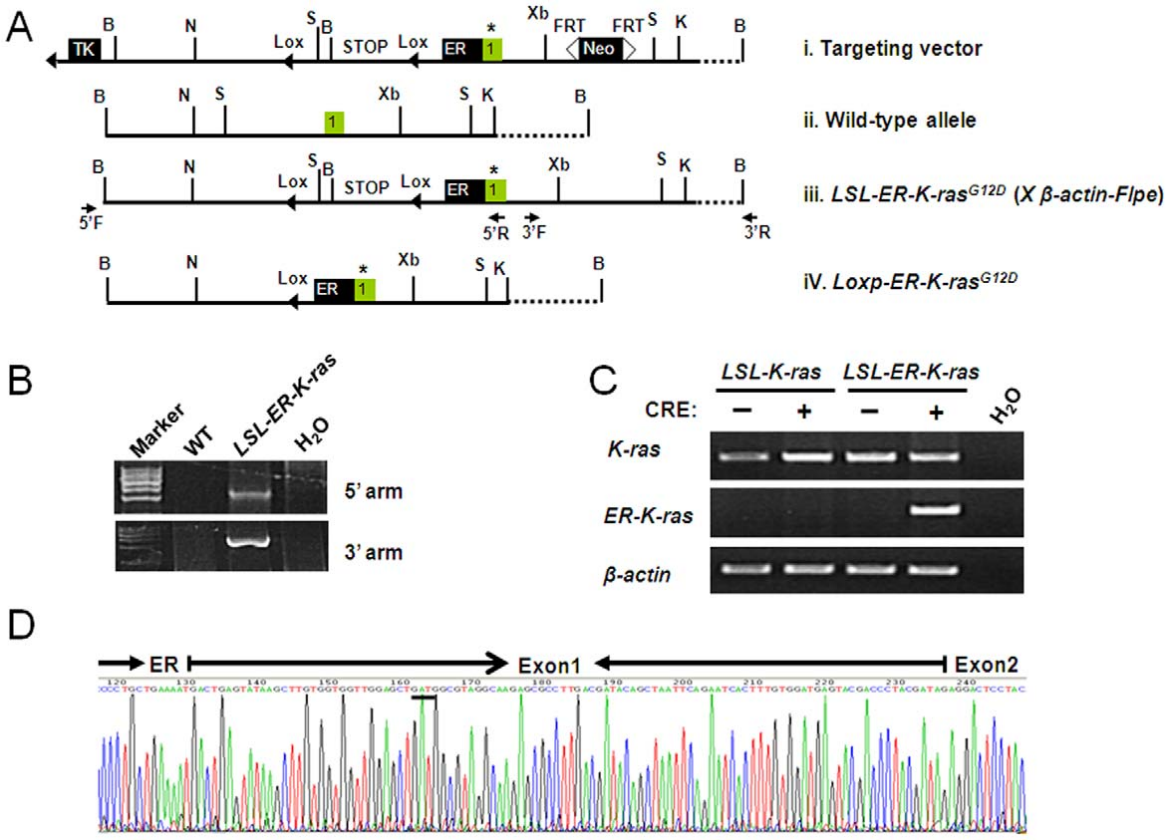
Establishment of a conditional regulatable K-ras^G12D^ knock-in allele. A) Construction of Loxp-Stop-Loxp-ER-K-ras^G12D^ mice allele. The Stop codon were flanked with two LoxP sites; the Neo cassette is flanked with two FRT sites; Positive selection cassette contains the gene for a viral Neo cassette and negative selection cassette contains the gene for a viral thymidine kinase (TK). The original mice were then crossed with β-actin-Flpe to delete the Neo cassette. Thymidine kinase (TK); *BamH* I (B); *Kpn* I (K); *Not* I (N); *Xba* I (Xb); *Sph* I (S); ER, estrogen receptor cDNA; *, GGT to GAT mutation at codon 12; B) The genomic DNA recombination was confirmed by the 5′arm and 3′arm PCR. C) Detection of mRNA level of either K-ras or ER-K-ras^G12D^ in indicated MEFs. The PCR primers used for *ER-K-ras^G12D^* amplification are located at *ER* region and *K-ras* exon 2 respectively. β-actin serves as control. (D) Sequencing results confirmed the expression of the ER-K-ras gene fusion in MEFs.

The *K-ras^G12D^* and *p53 L/L* mice were originally generously provided by T. Jacks (Cambridge, MA) and R. Depinho (Boston, MA), respectively. The *LSL-ER-K-ras^G12D^* mice allele was constructed by placing the estrogen receptor cDNA in front of the K-ras^G12D^ coding region as shown in Figure 1. The targeting vector carried a negative selection marker for diptheria toxin (DT), a positive selection marker for neomycin acetyltransferase (Neo) and *loxP* sites (black triangles). The restriction sites were *BamH* I (B); *Kpn* I (K); *Not* I (N); *Xba* I (Xb); *Sph* I (S). We electroporated embryonic stem cells and selected transformed cells by 3′ arm and 5′ arm PCR screening to identify 3 recombinants. Blastocyst injections were carried out with these different targeted clones and germline transmission was achieved. The *LSL-ER-K-ras^G12D^* mice were then crossed to *p53 L/L* mice to obtain *LSL-ER-K-ras^G12D^*, *p53L/L* mice. All mice were housed in a specific pathogen-free environment at Shanghai Institute of Biochemistry and Cell Biology and treated in strict accordance with protocols approved by the Institutional Animal Care and Use Committee of the Shanghai Institute of Biochemistry and Cell Biology, Chinese Academy of Sciences. These mice were treated with Adeno-Cre through nasal inhalation as described before [28]. After three weeks of nasal inhalation, we treated these mice with 4-hydroxy-tamoxifen with different doses daily via intraperitoneal injection. Mice were then sacrificed for pathological inspection. Some mice were stopped for tamoxifen treatment for one or two weeks and then sacrificed for pathological inspection. Genotyping primers for *p53L/L*, *LSL-K-ras^G12D^* and *LSL-ER-K-ras^G12D^* was listed as following: For *p53L/L*, forward primer: 5′-CACAAAAACAGGTTAAACCCAG-3′; reverse primer: 5′-AGCACATAGGAGGCAGAGAC-3′. For *LSL-K-ras^G12D^* and *LSL-ER-K-ras^G12D^*, same primers were used for genotyping. Forward primer: 5′-CTAGCCACCATGGCTTGAGT-3′; reverse primer:5′-TCCGAATTCAGTGACTACAGATG-3′.

**Figure 2:**
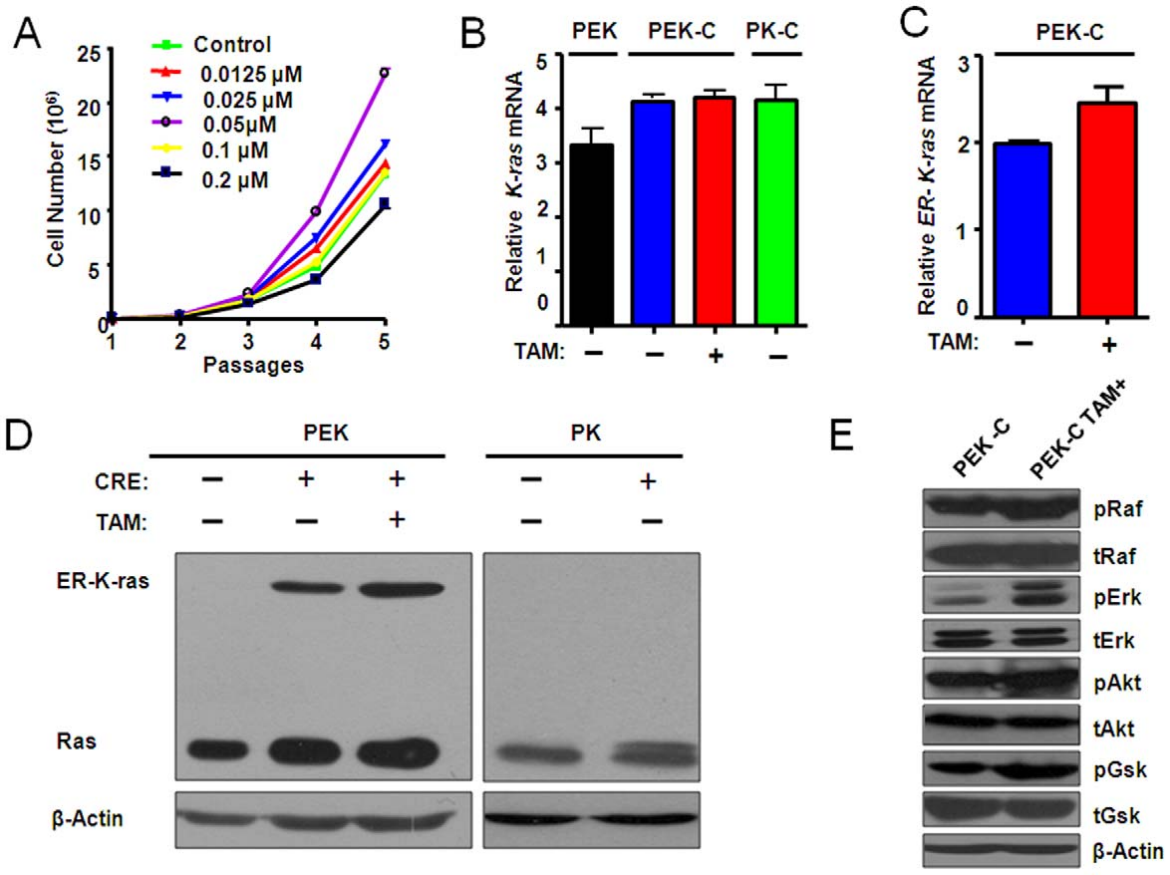
Treatment of tamoxifen at an optimal dose induced the activation of ER-K- ras^G12D^ and downstream signaling. A) The *P53−/−*, *ER-K-ras^G12D^* MEFs (PEK-C) derived from male embryo was counted for cell number after indicated dosage of tamoxifen treatment after 5 passages. B) Relative expression level of *K-ras* in indicated MEFs with or without tamoxifen treatment. PEK: *P53L/L*, *Loxp-Stop-Loxp ER-K-ras^G12D^*; PEK-C: *P53−/−*, *ER-K-ras^G12D^*; PK: *P53L/L*, *Loxp-Stop-Loxp-K- ras^G12D^*; PKC: *P53−/−*, *K- ras^G12D^*. (C) Detection of the ER-K-ras^G12D^ expression in PEK-C cells with or without 0.05 µM tamoxifen treatment. D) Detection of K-ras and ER-K-ras protein level in MEFs infected with or without adeno-Cre in the presence or absence of 0.05 µM tamoxifen treatment in indicated MEFs. β-actin serves as internal control. E) The activation of Ras effector PI3K pathway signaling was confirmed by western blot.

**Figure 3:**
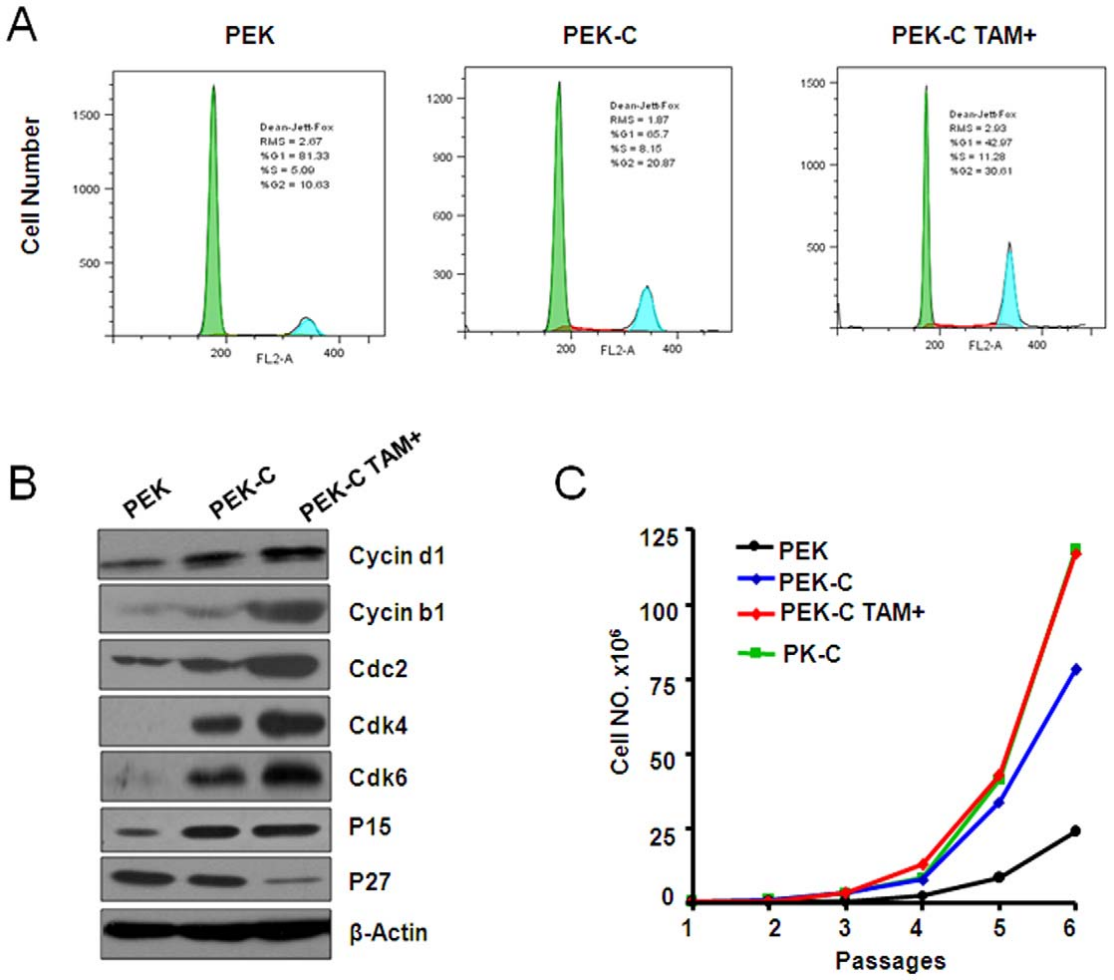
Induced activation of ER-K-ras^G12D^ by tamoxifen treatment promoted cell proliferation. A) The induced activation of ER-K-ras^G12D^ by tamoxifen treatment promoted cell entry into G2/M phase detected by FACS assay in PEK-C MEFs. B) Western blot analysis of cell cycle-related proteins in PEK-C MEFs with or without tamoxifen treatment. The PEK MEFs were used as control. β-actin serves as internal control for western blot. C) Cell number counting of indicated MEFs cultured for 6 passages with or without tamoxifen treatment.

**Figure 4:**
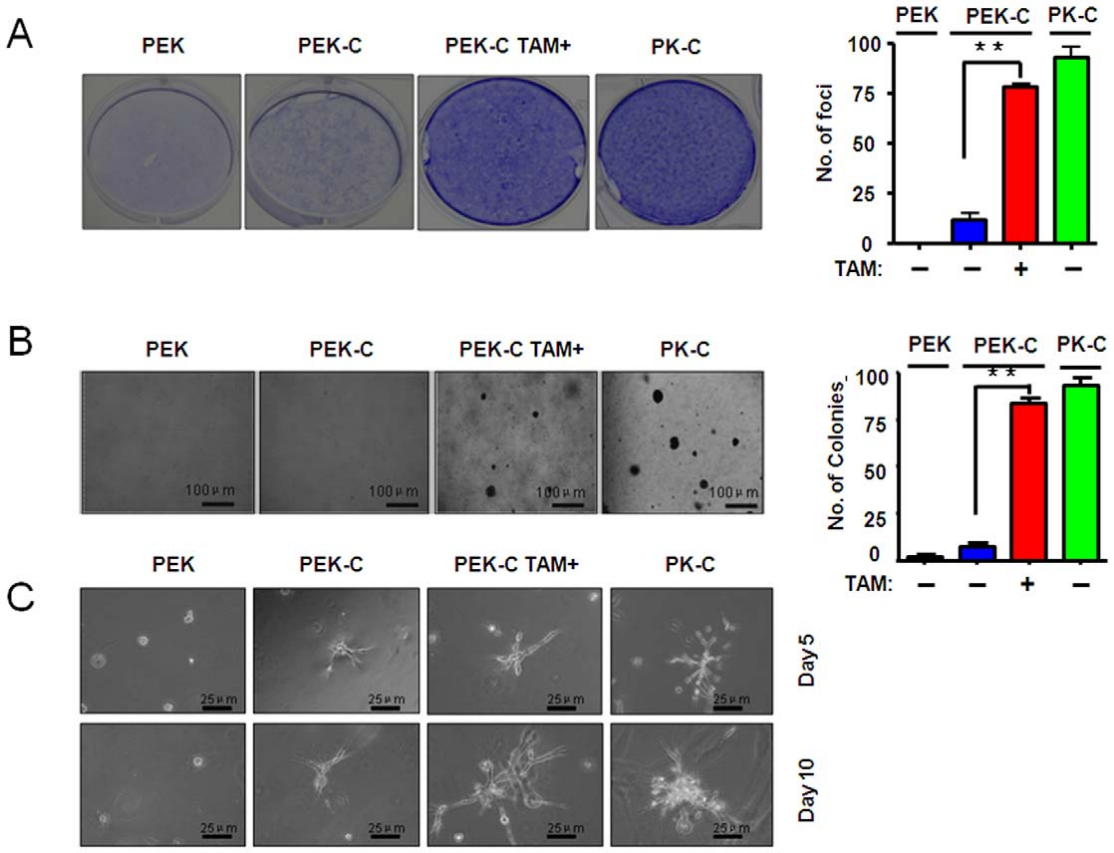
Induction of ER-K-ras^G12D^ activation by tamoxifen treatment is important for cell transformation and cell invasiveness *in vitro*. A) The foci formation assay were performed in PEK-C MEFs with or without tamoxifen administration. Bar graph indicated the number of foci observed in one well in 6-well plate. B) The PEK-C MEFs were assayed for anchorage-independent cell growth ability in soft agar in the absence or presence of tamoxifen administration. Bar graph indicated the colonies observed in soft agar per well in 6-well plate. C) The PEK-C MEFs were cultured in matrigel in the absence or presence of tamoxifen for indicated days and the cell invasiveness were assay as described in materials and methods.

**Figure 5:**
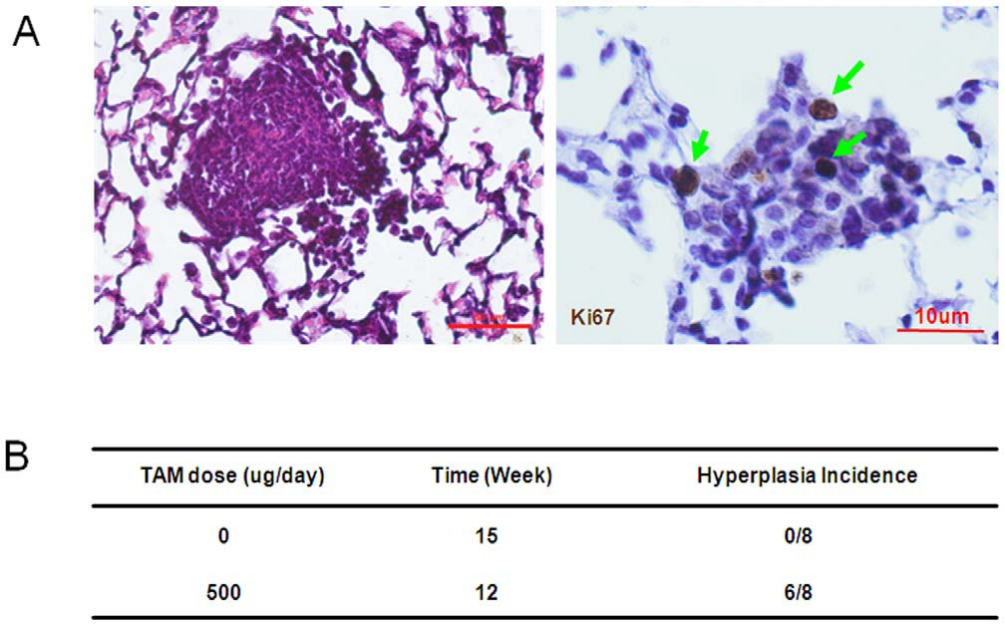
Continuous activation of ER-K-ras^G12D^ by 500 µg tamoxifen treatment induced lung hyperplasia and adenoma formation in P53 L/L, LSL-ER-K-ras mice. A) Continuous activation of ER-K-ras^G12D^ by 500 µg tamoxifen I.P. daily injection for 12 weeks is sufficient to drive lung adenoma formation in P53 L/L, LSL-ER-K-ras mice after adeno-Cre treatment. The adenoma were positive for Ki67 immunostaining. B) The incidence of lung hyperplasia and/or adenoma formation in *P53L/L*, *LSL-ER-K-ras^G12D^* mice was shown after adeno-Cre treatment in the absence or presence of tamoxifen treatment.

**Figure 6:**
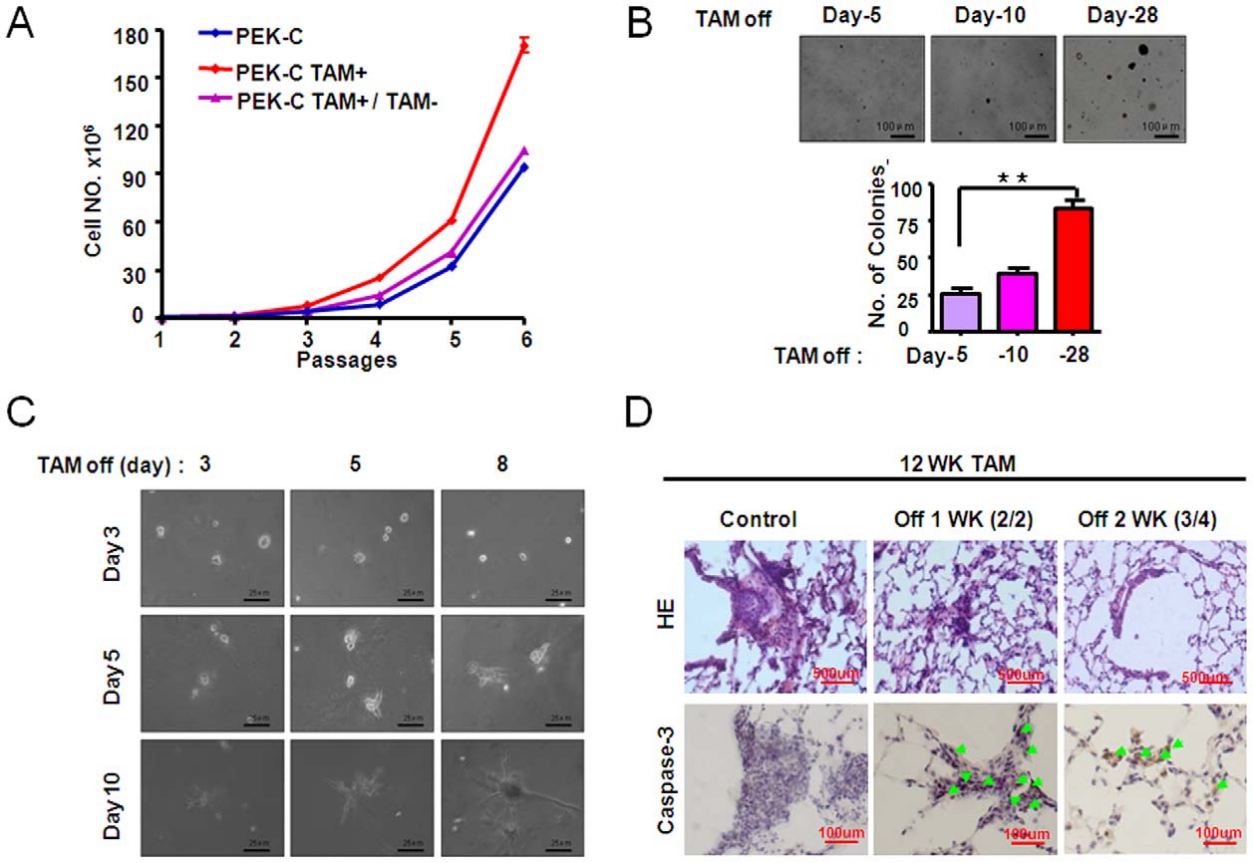
Sustained activation of ER-K-ras^G12D^ induced by tamoxifen treatment is important for cell proliferation, anchorage-independent cell growth, cell invasiveness and tumor maintenance. A) Cell number counting of indicated MEFs after withdrawal of tamoxifen after 6 passages in the presence of tamoxifen. The PEK-C MEFs always without tamoxifen treatment and the PEK-C MEFs always kept in tamoxifen were used as control. B) Tamoxifen were withdrawn from the PEK-C MEFs at day 5, 10 and 28 after continuous tamoxifen treatment in soft agar. C) Tamoxifen withdrawal from the PEK-C MEFs after indicated days of tamoxifen treatment in cell invasiveness assay in matrigel. D) Tamoxifen withdrawal for indicated time in the *P53L/L*, *LSL-ER-K-ras^G12D^* mice previously treated for 12 weeks of tamoxifen. The typical lung pathology and cleaved caspase-3 immunostaining were shown.

### Cell culture and cell proliferation assay

We generated MEFs from 13.5 post-coitum embryos and grew them in DMEM medium plus 10% fetal bovine serum (Biochrom, AG), penicillin and streptomycin. MEFs (∼4×10^5^) at 6-well plate were virally infected with Adeno-Cre (4×10^6^ CFU) overnight and then changed with fresh medium. The MEFs were then cultured for at least two more passages to get enough cells for various functional assays. Cell number were counted after each passage with or without tamoxifen treatment. Cells were also taken for PI staining and flow cytometry analysis for cell cycle.

### Biochemical assay

Total RNA was prepared and retro-transcribed into first-strand cDNA using RevertAid™ First Strand cDNA Synthesis Kit (Fermentas Cat: K1622). The cDNAs were then used for either regular PCR or real-time PCR on a 7500 Fast Real-Time PCR System (Applied Biosystems) using SYBR-Green Master PCR mix (Toyobo). *β-actin* served as internal control. Purification of gDNA in cells and mice tissues were previously described [29]. Primers used for RT-PCR were listed as following: For *K-ras^G12D^*, forward primer: 5′-TAAACTTGTGGTGGTTGGAG-3′; reverse primer: 5′-AACTCCTGAGCCTGTTTCGT-3′. For *ER- K-ras^G12D^*, forward primer: 5′-TCTACTTCATCGCATTCCTTG-3′; reverse primer: 5′-GGCACATCTTCAGAGTCCTTT-3′. For *β-actin*, forward primer: 5′-CGTTGACATCCGTAAAGACC-3′; reverse primer: 5′-AACAGTCCGCCTAGAAGCAC-3. Primers used for real-time PCR were listed as following: For *K-ras^G12D^*, forward primer: 5′-TGTGGATGAGTATGACCCTA-3′; reverse primer:5′-TACACAAAGAAAGCCCTCCCCA-3′. For *ER-K-ras^G12D^*, forward primer: 5′-TTTCCCTGCCACAGTCCC-3′; reverse primer: 5′-TCCATCAATTACTACTTGTTTCCTG-3′. For *β-actin*, forward primer: 5′-CAGCCTTCCTTCTTGGGTAT-3′; reverse primer: 5′-GGTCTTTACGGATGTCAACG-3′. Primers for detection of mouse gender using gDNA were listed as following: For *Sry*, forward primer: 5′- TGGTCCCGTGGTGAGAGGC -3′; reverse primer: 5′- TATGTGATGGCATGTGGGTTCC -3′. For *IL3*, 5′- GGGACTCCAAGCTTCAATCA -3′; reverse primer: 5′- TGGAGGAGGAAGAAAAGCAA -3′.

Total protein lysate were prepared by homogenization in protein loading buffer. Equal amounts of protein from each sample were separated by electrophoresis on an SDS-PAGE gel and transferred onto PVDF membranes. Western blot analysis was performed using following antibodies towards: Ras (Millipore, Cat: 05-1072), ER (Santa Cruz Cat: sc-543), Phospho-Raf (Cell Signaling, Cat: 9427), total Raf (Santa Cruz Cat: sc-5284), Phospho-Erk (Cell Signaling, Cat: 9180), total Erk (Santa Cruz Cat: sc-153), Phospho-Akt, Phospho-GSK (Cell Signaling, Cat: 9916), total Akt (Cell Signaling, Cat: 9272), total GSK (Bioworld Technology, Cat: BS1412), Cyclin d1, Cdk4, Cdk6, p15 (Cell Signaling, Cat: 9932), Cyclin B1 (Bioworld Technology, Cat: BS1392), Cdc2 (Bioworld Technology, Cat: BS1820) and β-actin (Santa Cruz, SC-1616).

### Cellular functional assay

MEFs with indicated genotype with or without tamoxifen (Sigma, Cat: 011M1682V) treatment were cultured in DMEM with 10% fetal bovine serum until 100% confluence. MEFs were changed with fresh medium every three days for 3 weeks before staining with 0.005% crystal violet. Foci formation were observed and the number of foci were counted.

As for anchorage-independent cell growth, MEFs were plated in 0.4% top agarose on top of a 1% agarose base supplemented with complete medium. MEFs were changed with fresh medium with or without tamoxifen every 3 days for about 4 weeks before staining with 0.005% crystal violet. The number of colonies (>0.5 mm) were counted under the microscope.

As for invasion assay, MEFs were cultured in matrigel (BD, Cat: 356234) in the presence or absence of tamoxifen treatment for 10 days. Photos with typical morphologic change were shown.

### Histopathological Analysis and Immunological Studies

Histopathological analysis were performed as described before [30]. Briefly, mice were sacrificed and lung tissues were inflated and fixed in 10% formalin, embedded in paraffin and sectioned for hematoxylin and eosin (HE) staining. For immunostaining, sections of 5 µm-thickness were cut from paraffin-embedded lung tissues, deparaffinized in xylene and rehydrated in ethanol. Deparaffinized sections were pretreated with 0.3% H_2_O_2_ (in absolute methanol) for 15 min at room temperature to block endogenous peroxidase activity. The slides were then heated in a microwave for three periods of 5 min high power, 2 min defrost and 20 min medium low power of microwave treatments. After re-cooling at room temperature for about 30 min, slides were rinsed twice in H_2_O for 3 min and then were treated for 2 h with normal non- immune goat serum albumin to block nonspecific staining. Sections were incubated overnight with Ki67 antibody (1∶100, Santa Cruz, SC-23900) and Cleaved Caspase-3 antibody (1∶300, Cell Signaling, Cat: 9661). The secondary biotinylated antibody was incubated for 15 min at 37°C. The slides were then stained using a diaminobenzidine tetrahydrochloride (DAB) detection kit (SP-9001), counterstained with haematoxylin and mounted with permount.

## Results

### Establishment of the knock-in mice with an endogenous conditional regulatable *LSL-ER-K-ras^G12D^* allele

We have made the conditional regulatable *LSL-ER-K-ras^G12D^* knock-in allele using the homologous recombination with the replacement of endogenous K-ras with a fragment containing of a 1.0 kb estrogen receptor cDNA fused to the K-ras^G12D^ (Fig. 1A). The mice were then crossed with β-actin-Flpe to delete the Neo cassette and the resultant mouse allele was named as *LSL-ER-K-ras^G12D^*. The homologous recombination were confirmed by the 5′arm and 3′arm PCR as described before (Fig. 1B) [31]. We obtained the *LSL-ER-K-ras^G12D^* mouse embryonic fibroblasts (MEFs) and infected them with Adeno-Cre. As expected we have detected the expression of *ER-K-ras^G12D^* at mRNA level (Fig. 1C), which was further confirmed by direct sequencing (Fig. 1D).

### Activation of ER-K-rasG12D and downstream signaling by tamoxifen treatment

We next determined if the *ER-K-ras^G12D^* MEFs are responsive to tamoxifen treatment. Tamoxifen is an anti-breast cancer agent and potentially toxic. We therefore titrated its dosage for the *ER-K-ras^G12D^* MEF treatment. Interestingly, we found that the proliferation rate of MEFs increased with increased tamoxifen dose from 0.0125 µM to 0.05 µM (Figure S2A–B). However, decreased cell proliferation was observed when an even higher dose of tamoxifen (from 0.1 µM to 0.2 µM) was given to MEFs (Figure S2A–B). Similar finding was observed in the *ER-K-ras^G12D^* MEFs with p53 deficient background (Fig. 2A and Figure S2C). Furthermore, both *ER-K-ras^G12D^* and *p53−/−*, *ER-K-ras^G12D^* MEFs (PEK-C) from either male or female has shown consistent response to tamoxifen treatment (Fig. 2A, Figure S1, Figure S2), suggesting a gender-independent pattern. Hereafter, we chose a dose of tamoxifen at 0.05 µM for further *in vitro* studies. P53 loss is commonly observed in human cancer [32]. Moreover, p53 deficiency results in the immortalization of MEFs and also significantly contributes to colony formation of K-ras^G12D^ MEFs in soft agar. For these reasons, we have therefore used the *p53−/−*, *ER-K-ras^G12D^* MEFs (refer to PEK-C) for our further studies. We compared the mRNA level of total K-ras in *p53L/L*, *LSL-ER-K-ras^G12D^* (PEK), *p53−/−*, *ER-K-ras^G12D^* (PEK-C) and *p53−/−*, *K-ras^G12D^* (PK-C) MEFs (Fig. 2B) as well as *LSL-K-ras^G12D^*, *LSL-ER-K-ras^G12D^*, *ER-K-ras^G12D^* MEFs with or without Adeno-Cre treatment (Figure S3A–C). No significant change of total K-ras mRNA and protein levels was observed among all MEFs with or without either Adeno-Cre or tamoxifen treatment (Fig. 2C–D). Using K-ras antibody, we have detected the ER-K-ras band with correct molecular weight (Fig. 2D). This was further confirmed by western blot using ER antibody (Figure S3D). Consistently, we have observed the phosphorylation of several Ras downstream effectors including Raf, Erk, Akt and Gsk after tamoxifen treatment (Fig. 2E).

### Inducible activation of ER-K-ras^G12D^ promotes cell proliferation

Abnormal cell proliferation is considered as an early event during tumorigenesis [33]. Tamoxifen administration significantly increased the cell division in MEFs indicated by an increase of cells at G2/M phase (Fig. 3A). Increase of cell cycle related proteins including Cyclin d1, Cdk4, Cdk6, p15, Cyclin b1, and Cdc2 were also observed (Fig. 3B). Conversely, p27 protein level decreased after tamoxifen treatment (Fig. 3B). We further checked the cell proliferation rate of PEK, PK-C, and PEK-C with or without tamoxifen treatment using cell number counting for 6 passages. We found that the tamoxifen treatment significantly increased cell proliferation in PEK-C MEFs. Actually, the growth rate of PEK-C MEFs treated with 0.05 µM tamoxifen is comparable to PK-C MEFs. Taken together, these data have demonstrated that ER-K-ras activation by tamoxifen treatment promoted cell proliferation *in vitro*.

### Activation of ER-K-ras^G12D^ is essential for cell transformation and cell invasiveness

We then determined if the inducible ER-K-ras activation promotes cell transformation, anchorage-independent cell growth and cell invasiveness in 3D cell culture. As expected, the PEK MEFs didn't form any foci. Tamoxifen treatment of PEK-C MEFs obviously promoted the foci formation, comparable to PK-C MEFs (Fig. 4A). The anchor-independent growth of PEK-C MEFs was also enhanced by activation of ER-K-ras^G12D^ after tamoxifen treatment (Fig. 4B). These data have demonstrated that the ER-K-ras^G12D^ activation is essential for cell transformation. Consistently, we found that tamoxifen administration significantly increased cell invasion in matrigel, suggesting that ER-K-ras^G12D^ activation by tamoxifen treatment may promote cell invasion (Fig. 4C).

### Continuous activation of ER-K-ras^G12D^ induced lung hyperplasia

RAS mutant play a pivotal role in neoplastic transformation and tumorigenesis [13], [19], [34], [35], [36]. To test the *in vivo* function of ER-K-ras in lung tumorigenesis, we first tried tamoxifen treatment at different doses in mice. All the *p53L/L*, *LSL-ER-K-ras* ^G12D^ mice were given Adeno-Cre at 6∼8 week-old. After three weeks of Adeno-Cre treatment, these mice were then given tamoxifen daily via intraperitoneal (IP) injection. Our pilot experiment has used a series of different tamoxifen doses at 0, 100, 250, 500, 1000 µg per mouse. All the mice without tamoxifen treatment showed normal lung histology (Fig. 5B and Figure S4). Mice with low doses of tamoxifen treatment at 100 or 250 µg displayed normal lung pathology, similar to those with a higher dose of tamoxifen at 1000 µg (Figure S4). Interestingly, mice receiving tamoxifen at 500 µg were found to have lung hyperplasia as early as after 5-week treatment (Figure S4). Lung adenomas, positive for Ki-67 staining, arose at 8 weeks post tamoxifen treatment (Fig. 5A). These data suggested that an optimal dose of tamoxifen is sufficient to drive lung epithelial cell proliferation, hyperplasia and early tumor formation.

### Continuous activation of ER-K-ras^G12D^ is important for tumor maintenance

To further test the potential role of K-ras mutant in tumor maintenance, we have performed both *in vitro* and *in vivo* studies. As we have shown above, tamoxifen treatment significantly promoted PEK-C MEFs proliferation after several passages (Fig. 3C). However, after tamoxifen withdrawal, these MEFs obviously reduced the proliferation rate in comparison with PK-C MEFs (Fig. 6A). Similarly, tamoxifen withdrawal resulted in a dramatic decrease of the colony formation ability and cell invasiveness of these MEFs (Fig. 6B–C).

Our data have shown that continuous tamoxifen treatment at an optimal dose for 12 weeks consistently drove lung hyperplasia formation in *p53L/L*, *LSL-ER-K-ras^G12D^* after Adeno-Cre treatment (Fig. 5). We then stopped tamoxifen treatment afterwards for either 1 week or 2 week. Pathological inspection have shown that the lung hyperplasia significantly decreased in mouse lungs even after one week of tamoxifen withdrawal (Fig. 6D). The tumor regression is accompanied by apoptosis indicated by increased cleaved caspase-3 immunostaining (Figure 6D). The mouse lungs were largely normal after two weeks of tamoxifen withdrawal (Fig. 6D), suggesting that continuous activation of K-ras mutant is important for tumor maintenance.

## Discussion

Temporal controlling K-ras activity *in vitro* and *in vivo* is of great importance for dissection of its function in tumorigenesis. We have here constructed a conditional inducible oestrogen receptor-K-ras^G12D^ (*ER-K-ras^G12D^*) knock-in mice allele that allows us to temporally switch on or off the K-ras oncogenic mutant through the tamoxifen administration. This allele is able to be regulated by tamoxifen treatment at an optimal dosage *in vitro* and *in vivo*. Our data have demonstrated that the deregulation of K-ras activity plays an important role in neoplastic transformation as well as tumor maintenance.

Several animal models have been generated to study the role of *K-ras* mutant in tumorigenesis [28], [33], [37], [38]. Animal model with oncogenic alleles of *K-ras^LA^* was initially constructed via homologous recombination [37]. The *K-ras^LA^* mice have developed a variety of tumor types including lung neoplastic transformation [37]. However, an early mortality was caused by an overwhelming number of predominantly early-stage lung lesions which limits the capacity for tumor progression [37]. A transgenic mouse allele with doxycycline-inducible K-ras4B^G12D^ expression was established under the control of rat Clara cell secretory protein CCSP promoter [38]. Although this mouse allele has been proved to be very helpful in the regulation of K-ras expression with time, it's a transgenic line with potentially multiple copies and uncertain genomic insertion sites which may influence the function of K-ras mutant. A significantly improved animal model was generated by placing a Loxp-stop-Loxp cassette fragment in front of K-ras^G12D^ coding region, in which the K-ras mutant doesn't express until the removal of stop codon by either Adeno-Cre treatment or tissue-specific CRE transgene expression [28], [33]. This model has been widely used for cancer studies, especially for the study on tumor initiation and progression process [4], [7], [29], [39], [40]. With this model, once the *K-ras* mutant is activated, it can't be turned off, which may limit it's usage in studying tumor maintenance. Our animal model with ER knock-in into K-ras gene allele allows us to temporally control the K-ras activity via tamoxifen administration, which makes it possible to dissect the role of K-ras mutant at different stages of tumorigenesis.

Tamoxifen is an anti-estrogen commonly used in the treatment of breast cancer. Previous studies have showed that a dosage range between 10 nM to 1 µM is able to inhibit cell growth [41], [42]. Therefore, the tamoxifen stimulation of ER-fusion protein activation may somehow be dependent on the dosage. Consistently, a 4OHT concentration-dependent induction of fusion protein activity were also observed previously [1], [24], [25], [26], [27], [43], [44]. In our study, we have observed that a dose of tamoxifen at 0.05 µM works effectively for stimulating the activation of ER-K-ras^G12D^ and cell proliferation. However, we are not certain about the optimal dosage of tamoxifen for *in vivo* treatment. As we know, it usually takes about more than 20 weeks for us to observe tumor nodules on lung surface in *K-ras^G12D^* mouse model [45] and our unpublished data). In contrast, simultaneous p53 deficiency with K-ras activation significantly facilitates lung tumor progression. Most *K-ras^G12D^* mice with p53 deficiency develop large tumors on lung surface at 10 weeks post Adeno-Cre treatment [46]. To shorten the potential tamoxifen treatment duration and make our *in vivo* study more practical, we have therefore tested the tamoxifen dosage in P53L/L, *ER-K-ras^G12D^* mice after Adeno-Cre treatment. Interestingly, we have found that tamoxifen treatment at 500 µg is sufficiently to drive lung hyperplasia in P53L/L, *ER-K-ras^G12D^* mice. However, the tumor latency is much longer than that of the P53L/L, *K-ras^G12D^* mouse model. It takes about 12 weeks of tamoxifen treatment for the P53L/L, *ER-K-ras^G12D^* mice to develop lung tumors. One possibility for this could be the low level of ER-K-ras^G12D^ activation by tamoxifen treatment at 500 µg. Future study is definitely necessary to optimize the tamoxifen dosage to achieve the full-activation of ER-K-ras^G12D^.

In sum, we have here constructed a mice allele with regulatable *K-ras* and demonstrated that the activation of K-ras is important for tumor formation as well as tumor maintenance. This animal model may provide a useful platform for further detailed characterization of the role of *K-ras* during different stages of tumorigenesis.

## Supporting Information

Figure S1 **Genotyping of MEFs.** A) Genotyping of *Loxp-Stop-Loxp-K-ras^G12D^* before and after Adeno-Cre treatment in different MEFs. B) Genotyping of *p53* MEFs before and after Adeno-Cre treatment in different MEFs.
(TIF)

Figure S2 **0.05 µM tamoxifen treatment induced Ras^G12D^ activation both in male and female** ***ER-K-ras^G12D^*** **MEFs (A and B) and in PEK-C female MEFs (C).**
(TIF)

Figure S3 **The expression of** ***K-ras*** **and** ***ER-K-ras*** **on mRNA and protein level in different MEFs.** A) The expression of *K-ras* has no obvious change on mRNA level in *Loxp-Stop-Loxp-K-ras^G12D^* MEFs and Loxp-Stop-Loxp-ER-K-ras^G12D^ with or without Adeno-Cre treatment. B-C) The expression of *ER-K-ras^G12D^* has no obvious change on mRNA level in *ER-K-ras* MEFs in the absence (B) or in the presence (C) of tamoxifen treatment. D) Detection of ER and ER-K-Ras protein level in MEFs infected with or without Adeno-Cre in the presence or absence of 0.05 µM tamoxifen treatment in indicated MEFs. β-actin serves as internal control.
(TIF)

Figure S4 **500 µg per mouse per day tamoxifen treatment could induce ER-K-ras^G12D^ activation and lead lung hyperplasia (A and B).**
(TIF)
