# Correction: Temporal Dissection of K-ras^G12D^ Mutant *In Vitro* and *In Vivo* Using a Regulatable K-ras^G12D^ Mouse Allele

**DOI:** 10.1371/annotation/0671c124-a263-49e9-9c23-421fd125db2c

**Published:** 2012-07-10

**Authors:** Zuoyun Wang, Yan Feng, Nabeel Bardessy, Kwok-Kin Wong, Xin-Yuan Liu, Hongbin Ji

The third author's name was spelled incorrectly. The correct name is: Nabeel Bardeesy.

The correct citation is: Wang Z, Feng Y, Bardeesy N, Wong K-K, Liu X-Y, et al. (2012) Temporal Dissection of K-rasG12D Mutant In Vitro and In Vivo Using a Regulatable K-rasG12D Mouse Allele. PLoS ONE 7(5): e37308. doi:10.1371/journal.pone.0037308

