# Supplementary figures and images for: Temporal Dissection of K-rasG12D Mutant In Vitro and In Vivo Using a Regulatable K-rasG12D Mouse Allele
Source: PLoS One. 2012 May 11;7(5):e37308. doi: 10.1371/journal.pone.0037308 (PMC3350485; doi:10.1371/journal.pone.0037308)

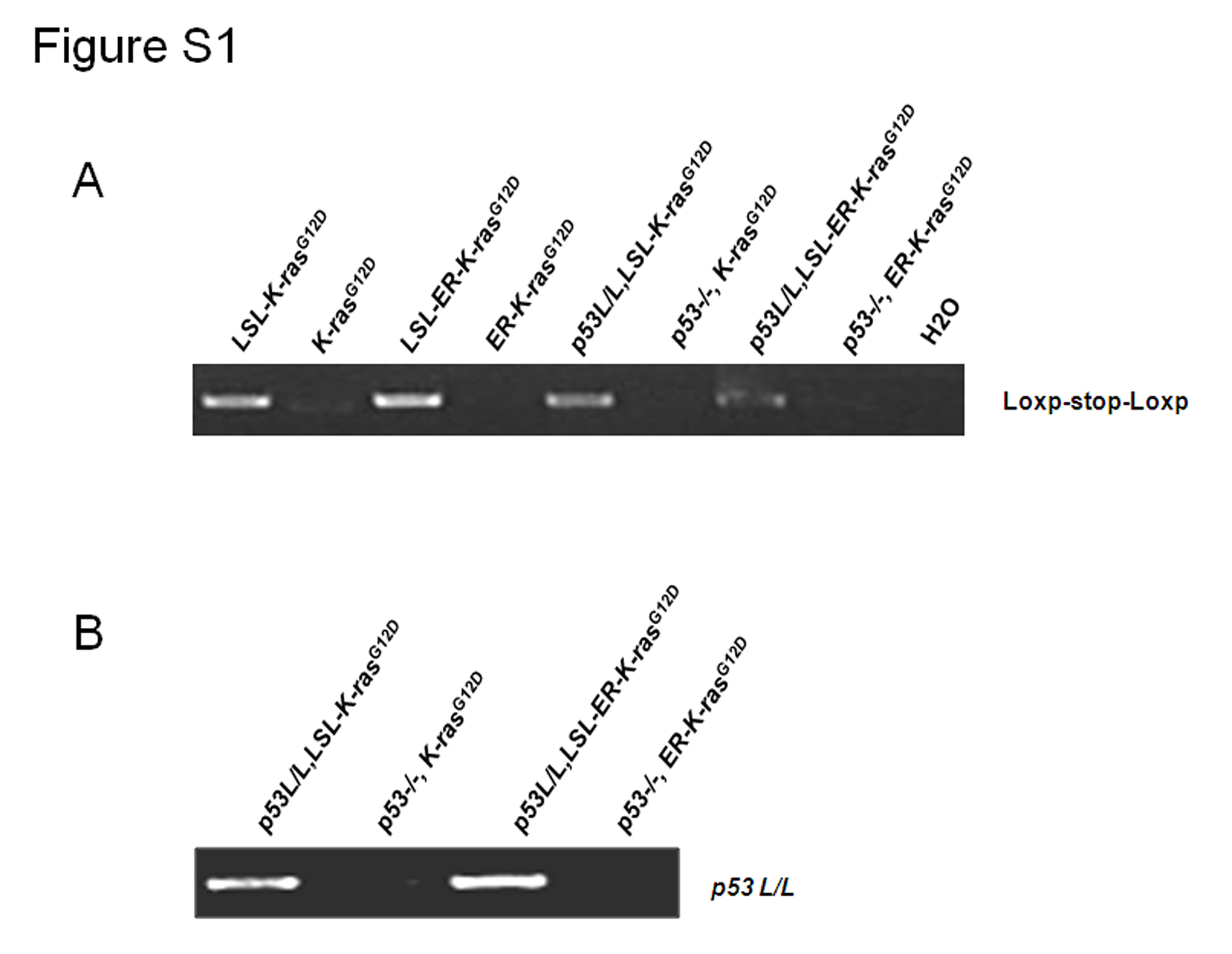

Supplement: Figure S1 — Genotyping of MEFs. A) Genotyping of Loxp-Stop-Loxp-K-rasG12D before and after Adeno-Cre treatment in different MEFs. B) Genotyping of p53 MEFs before and after Adeno-Cre treatment in different MEFs. (TIF) [file pone.0037308.s001.tif]

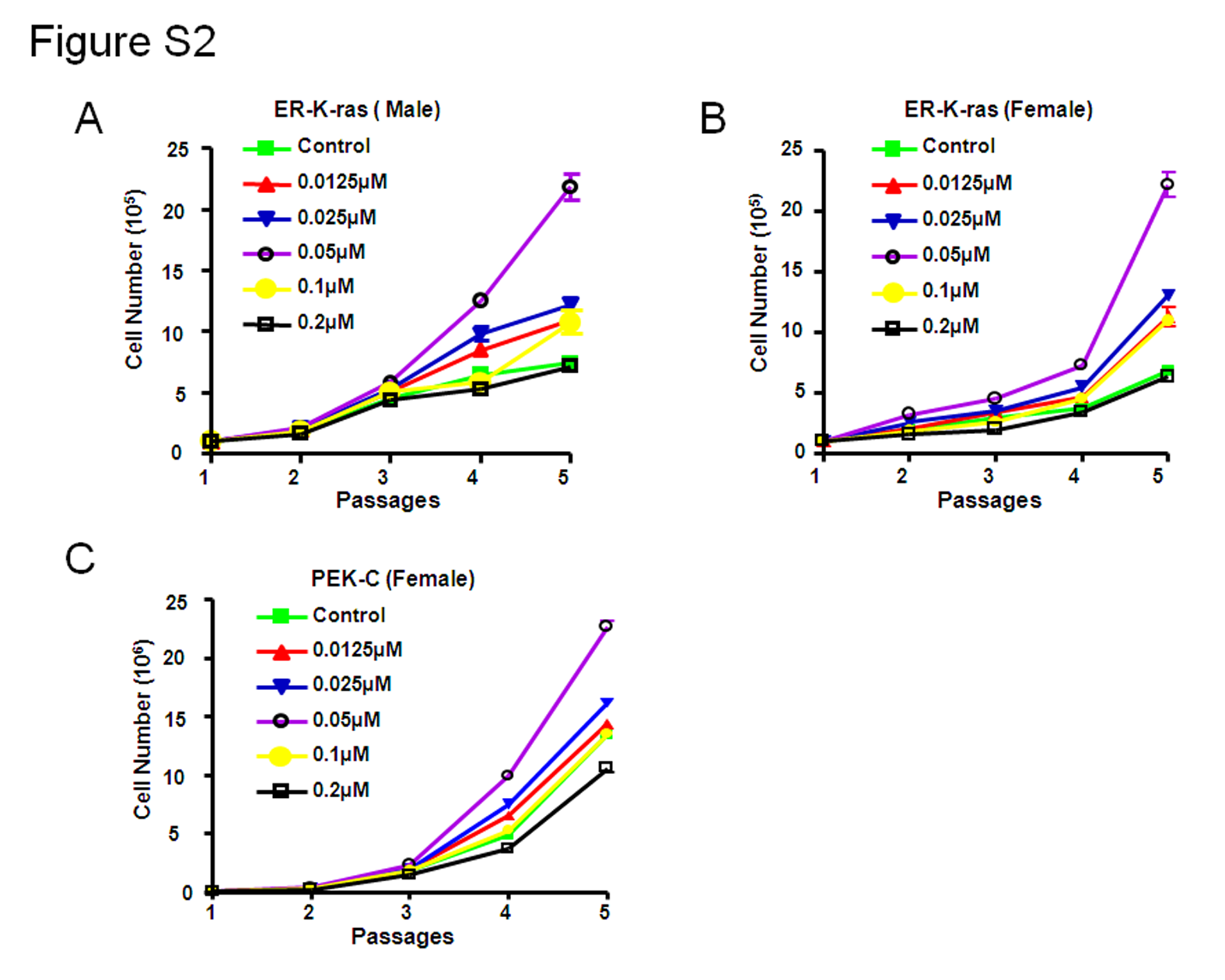

Supplement: Figure S2 — 0.05 µM tamoxifen treatment induced RasG12D activation both in male and female ER-K-rasG12D MEFs (A and B) and in PEK-C female MEFs (C). (TIF) [file pone.0037308.s002.tif]

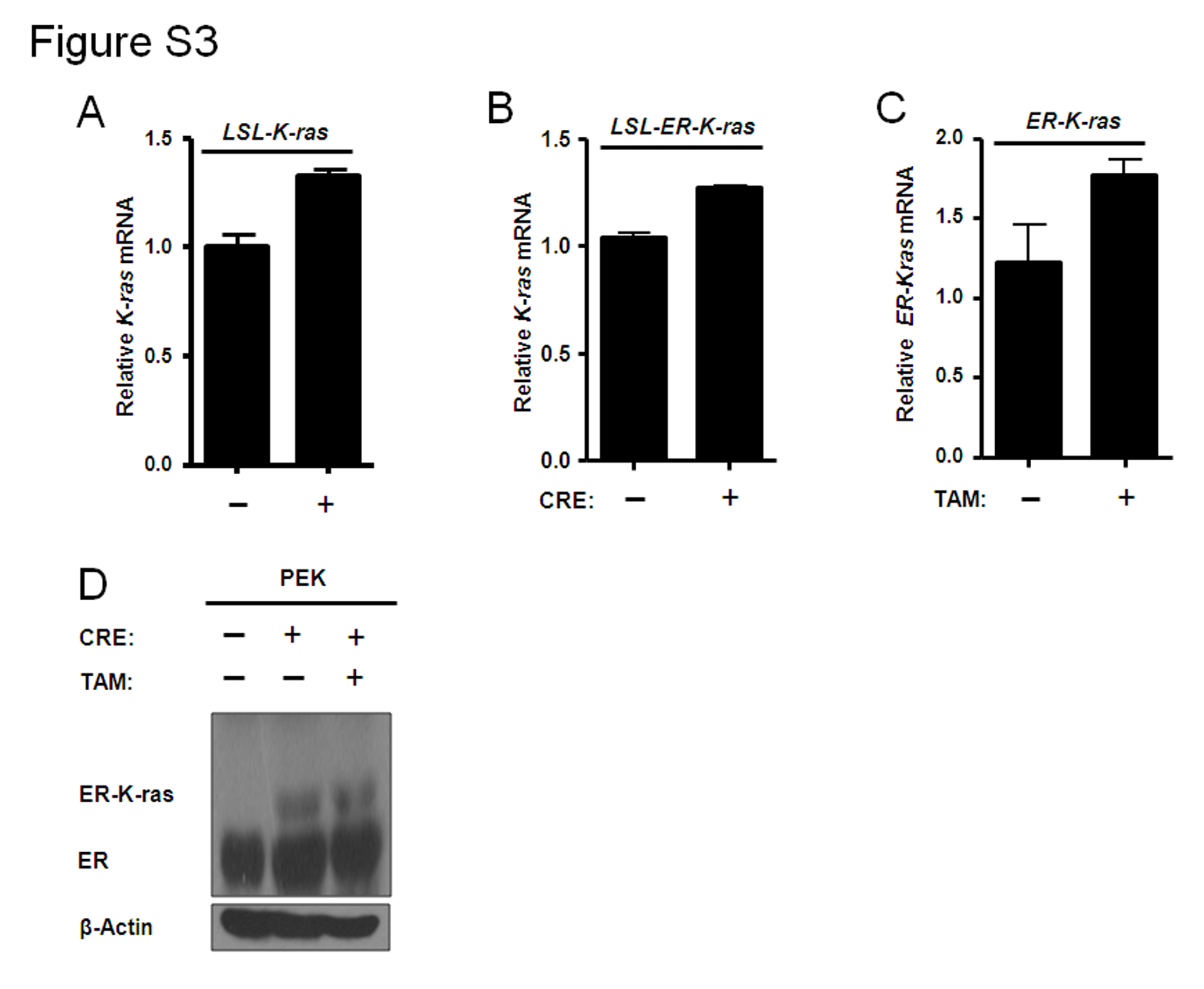

Supplement: Figure S3 — The expression of K-ras and ER-K-ras on mRNA and protein level in different MEFs. A) The expression of K-ras has no obvious change on mRNA level in Loxp-Stop-Loxp-K-rasG12D MEFs and Loxp-Stop-Loxp-ER-K-rasG12D with or without Adeno-Cre treatment. B-C) The expression of ER-K-rasG12D has no obvious change on mRNA level in ER-K-ras MEFs in the absence (B) or in the presence (C) of tamoxifen treatment. D) Detection of ER and ER-K-Ras protein level in MEFs infected with or without Adeno-Cre in the presence or absence of 0.05 µM tamoxifen treatment in indicated MEFs. β-actin serves as internal control. (TIF) [file pone.0037308.s003.tif]

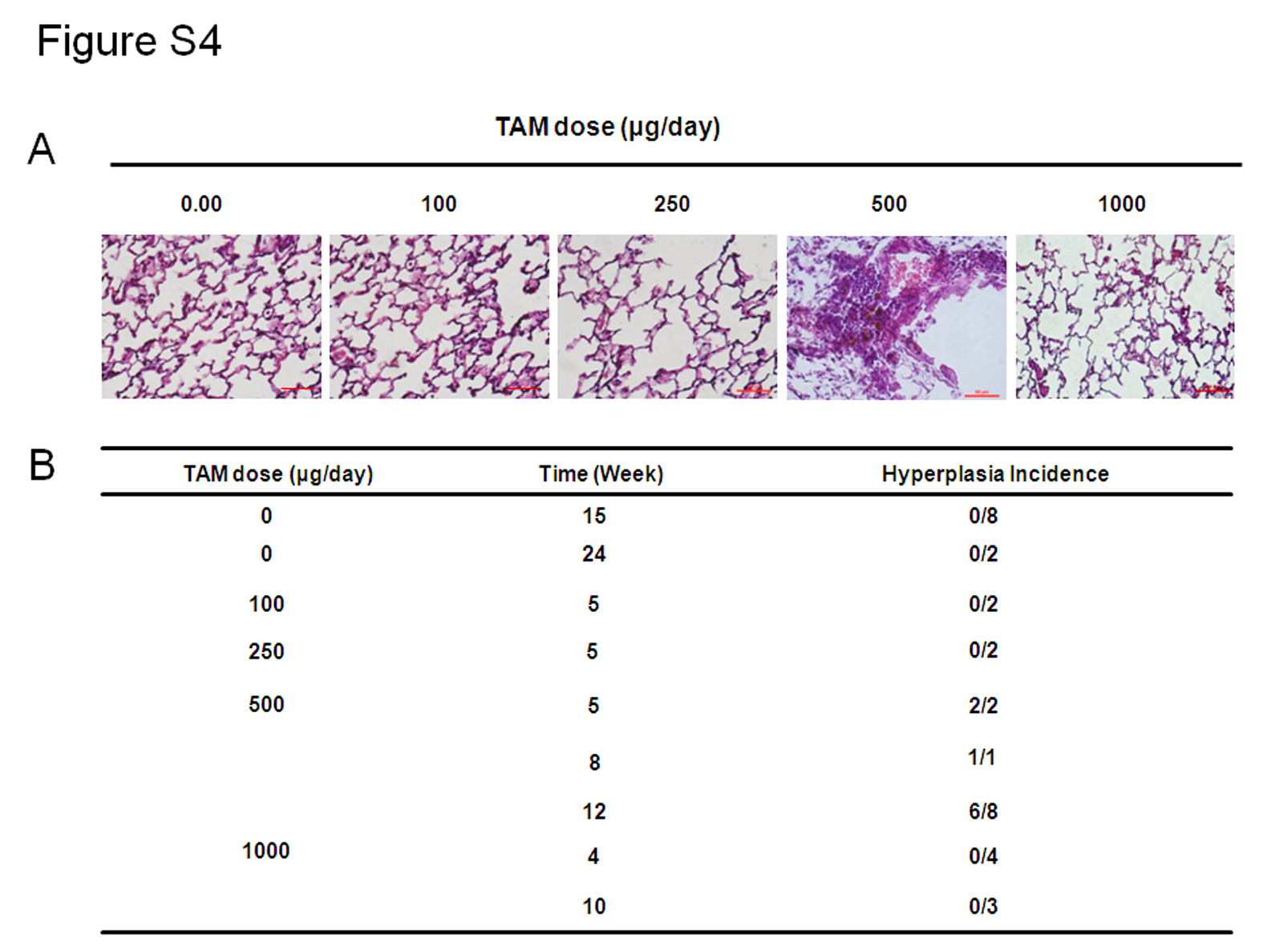

Supplement: Figure S4 — 500 µg per mouse per day tamoxifen treatment could induce ER-K-rasG12D activation and lead lung hyperplasia (A and B). (TIF) [file pone.0037308.s004.tif]
